# On the relations between letter, word, and sentence-level processing during reading

**DOI:** 10.1038/s41598-022-22587-1

**Published:** 2022-10-22

**Authors:** Brice Brossette, Jonathan Grainger, Bernard Lété, Stéphane Dufau

**Affiliations:** 1 Laboratoire d’Étude des Mécanismes Cognitifs, Lyon 2 University, Lyon, France; 2 Laboratoire de Psychologie Cognitive, CNRS and Aix-Marseille University, 3 Place Victor Hugo, 13331 Marseille, France; 3 Institute for Language Communication and the Brain, Aix-Marseille University, Marseille, France; 4 Queensland Brain Institute, University of Queensland, Brisbane, QLD Australia

**Keywords:** Human behaviour, Visual system

## Abstract

Much prior research on reading has focused on a specific level of processing, with this often being letters, words, or sentences. Here, for the first time in adult readers, we provide a combined investigation of these three key component processes of reading comprehension. We did so by testing the same group of participants in three tasks thought to reflect processing at each of these levels: alphabetic decision, lexical decision, and grammatical decision. Participants also performed a non-reading classification task, with an aim to partial-out common binary decision processes from the correlations across the three main tasks. We examined the pairwise partial correlations for response times (RTs) in the three reading tasks. The results revealed strong significant correlations across adjacent levels of processing (i.e., letter-word; word-sentence) and a non-significant correlation between non-adjacent levels (letter-sentence). The results provide an important new benchmark for evaluating computational models that describe how letters, words, and sentences contribute to reading comprehension.

## Introduction

**Figure 1:**
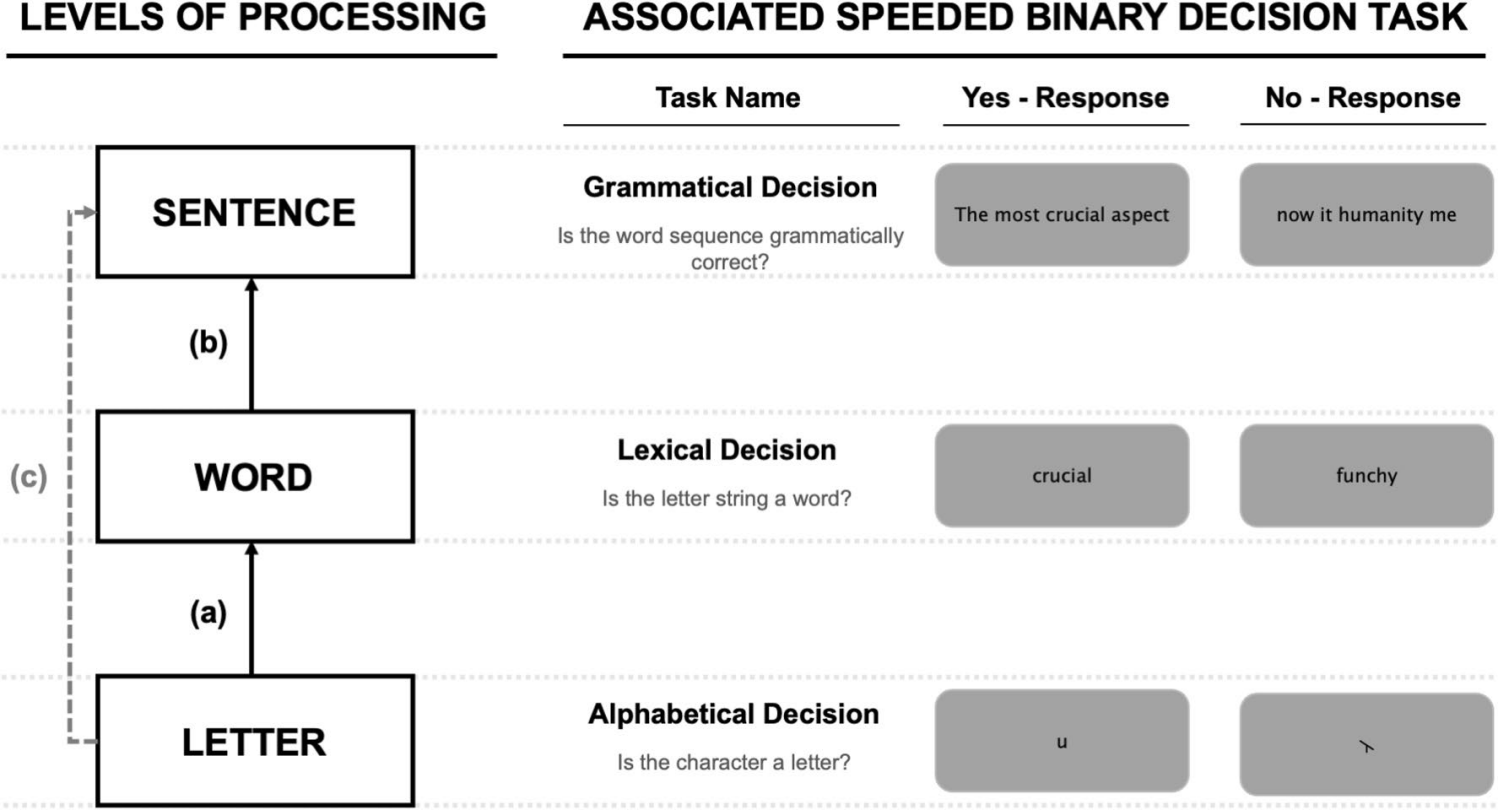
A hierarchical model of reading with letter, word, and sentence level representations. The figure illustrates the central hypothesis to be tested in the present study—that letters connect with words (a) and words with sentences (b), but letters do not directly connect with sentences (c).

For readers of a language written with an alphabetic script, fluent reading behavior essentially involves extracting information about letter identities and their positions to identify words and word order, and from there to construct a sentence-level representation for comprehension. Although this deliberate over-simplification ignores the well-established roles played by phonology^1,2^ and morphology^3^ in skilled reading, and also the higher-level processes involved in text comprehension^4^, we believe that it accurately highlights three key component processes involved in transforming visual features into meaning during reading. In the present study we investigate, for the first time, the processing interactions between these three levels. Prior research has either focused on a single level of processing, or the interactivity between two levels (letter-word or word-sentence: these interactions are respectively illustrated by path (a) and path (b) in Fig. 1). We note nevertheless that, contrary to adult studies, developmental studies of reading typically use several tasks such as reading aloud, rapid automatized naming (RAN), reading comprehension (e.g., Landerl et al.^5^, Lefèvre et al.^6^, Muter et al.^7^). Focusing on letter, word, and sentence processing allowed us to employ three very comparable tasks when measuring the processing at each of these levels. These are the alphabetic decision task^8^, the lexical decision task^9^, and the grammatical decision task^10^. All three tasks are speeded binary decision tasks with a clearly defined target category and well-defined criteria for constructing non-target stimuli (see examples in Fig. 1).

In order to investigate interactions between these three key component processes, in the present study participants performed alphabetic, lexical, and grammatical decision tasks, and we examined correlations between performance in each of the three tasks with the aim to evaluate the interdependence of processing across each of the three putative levels being examined (letter, word, sentence). We then used the obtained correlations to examine possible differences in the interdependencies between processing at the letter, word, and sentence levels. Thus, it is possible that word recognition is highly constrained by letter-level processing, whereas a similar contingency might not be so strong for word and sentence-level processing. It is also theoretically interesting to ask whether letter-level processing can directly constrain sentence-level processing. For example, in the OB1-reader model of sentence reading^11^, word length in number of letters has a direct impact on how different word identities are assigned to a specific position in a line of text.

The overarching theoretical framework guiding this research is inspired by the interactive-activation model^12–15^. Here we apply this framework in its simplest form, referring uniquely to the different levels of processing that are postulated therein, and leaving aside some central assumptions about the nature of processing (e.g., parallel, cascaded, interactive). With respect to the present study, the key aspect of this framework is that processing proceeds hierarchically, from one level to the next. This simple hierarchical model of reading is illustrated in Fig. 1. We immediately acknowledge the deliberately over-simplistic nature of this architecture, which ignores the well-established role played by phonology and morphology in reading (e.g., Grainger^16^). This over-simplification was necessary in order to generate clear predictions with respect to potential relations in performance across the different reading tasks.

This hierarchical architecture makes clear predictions about how processing at a given level should influence processing at the other levels. If letter-level processing is a key component of word recognition, and if the alphabetic decision task accurately reflects letter-level processing and the lexical decision task accurately reflects word-level processing, then the correlation between performance in these two tasks should be very high. The same reasoning holds for word-level and sentence-level processing, assuming that the grammatical decision task accurately reflects processing at the sentence level. Examining the cross-task correlations between alphabetic decision and lexical decision on the one hand, and lexical decision and grammatical decision on the other, will allow us to estimate the relative contributions of the different component processes to the overall task of reading.

Prior research has provided evidence for interesting parallels between letter-word processing on the one hand, and word-sentence processing on the other. The “word superiority effect”^17–19^ refers to the higher accuracy in single letter identification when the target letter is presented in a word (e.g., the letter B in TABLE) compared with a pseudoword (e.g., the letter B in PABLE). More recently, a “sentence superiority effect”^20,21^ has been reported whereby identification of a single word target is better when that word is presented in the context of a correct sentence (e.g., target BOY in the sentence: “the boy runs fast”) compared with identification of the same word at the same position in an ungrammatical sequence (e.g., “runs boy fast the”). A further point of comparison, of particular interest for the present work, concerns transposed-letter effects observed in the lexical decision task (it is harder to classify a nonword formed by transposing two letters in a real word (e.g., “gadren” derived from “garden”) compared with nonwords formed by substituting two letters (e.g., “gatsen”)^22–24^, and transposed-word effects in the grammatical decision task (it is harder to reject ungrammatical sequences formed by transposing two words in a correct sentence (e.g., “The white was cat big”) compared with sequences that cannot be transformed into a correct sentence by transposing any two words (e.g., “The white was cat slowly”)^10,25^. The important point, with respect to the present study, is the fact that similar phenomena can be observed across the letter-word and the word-sentence interfaces.

In the present study we used three tasks that have been previously applied to study letter, word, and sentence-level processing. Crucially, all three tasks require a speeded binary decision as to whether or not the target stimulus belongs to a well-defined category (letters, words, sentences) relative to a background of stimuli that are designed make the discrimination difficult. The present study was motivated by the hypothesis that these three tasks could provide comparable insights into letter, word, and sentence-level processing. The alphabetic decision task involves speeded letter vs. non-letter discrimination. In the present study we opted to use the pseudo-letters provided by Vidal et al.^26^ as representing the best comparison relative to the pseudowords that are typically used in the lexical decision task. Direct proof that this task does reflect letter-level processing was provided by New and Grainger^27^, where robust effects of letter frequency were reported. The lexical decision task is quite simply the most widely used task to study single word recognition. The speeded version of the grammatical decision task is a more recent invention. Traditionally, grammaticality judgements, or well-formedness judgments, have been used by linguists in paper-and-pencil investigations of the nature of syntactic knowledge. Mirault et al.^10^ used a speeded binary decision version of grammaticality judgments (termed the “grammatical decision task” by Mirault and Grainger^28^) where they manipulated the nature of the ungrammatical sequences. Here we used the grammatical decision task as the sentence-level equivalent of lexical decisions to words and alphabetic decisions to letters. Thus, the ungrammatical sequences were chosen to be sentence-like in the same way that the pseudo-words were word-like, and the pseudo-letters were letter-like.

In the present study we set-out to examine cross-task correlations with performance in the three tasks described above with the same group of participants. This is the first time that such cross-task correlations have been examined across different levels of processing. Because the amount of shared processing is expected to be greater between two adjacent levels (letter-word; word-sentence) than between two non-adjacent levels (letter-sentence), we predicted that adjacent levels of processing (letter-word; word-sentence) should reveal stronger correlations than the correlation for non-adjacent levels of processing (letter-sentence). Participants were also tested in a speeded animal / non-animal decision task with drawings of familiar animals and inanimate objects. The aim here was to use performance on this non-reading task to partial out the contribution of common binary-decision making mechanisms in driving correlations across the three reading tasks. This specific task, compared with a simple stimulus detection task for example, has the advantage of involving greater depth of processing while using non-linguistic stimuli. That is, the animal decision task involves making speeded binary decisions based on semantic information (i.e., “animalness”) extracted from visual information, and we considered this to be the best average approximation to the amount of processing involved in the three reading tasks. Although alphabetic decision likely does not involve semantic information, we believe that the animal decision task is a good comparison point for this task to the extent that both tasks involve speeded binary decisions to simple visual stimuli. We nevertheless note that we might not have come up with the most appropriate baseline (non-reading) task. It will therefore be important for future research to examine how the use of a different task here (or different tasks) might impact on the obtained results.

## Results

The dataset consisted of 29,520 observations: 4920 for Alphabetical Decision Task (hereafter, ADT), 9840 for Lexical Decision Task (LDT), 9840 for Grammatical Decision Task (GDT), and 4920 for Non-Reading Task (NRT). Firstly, we provide descriptive statistics on RTs and error rates to give an overview of performance in each task. Condition means for RTs and error rates are shown in Table 1. The main analysis of the lexical decision task included all words that were tested, including morphologically complex words. Additional analyses limited to only morphologically simple words showed exactly the same pattern.

**Table 1:** Mean RTs (in milliseconds) for correct “yes” and “no” responses (standard errors in parentheses), and percentage of errors for yes and no-responses for each task.

	Task			
	NRT	ADT	LDT	GDT
**Response times**				
Mean RTs for yes-responses	536 ms (2.73)	499 ms (2.43)	613 ms (2.39)	1098 ms (5.92)
Mean RTs for no-responses	555 ms (2.92)	515 ms (2.39)	693 ms (2.92)	1345 ms (7.70)
**Error rates**				
Percentage of errors for yes-responses	2.48%	2.93%	4.04%	6.30%
Percentage of errors for no-responses	1.59%	1.59%	3.07%	16.4%

*NRT* non-reading task, *ADT* alphabetic decision task, *LDT* lexical decision task, *GDT* grammatical decision task.

### Response times (RTs)

**Figure 2:**
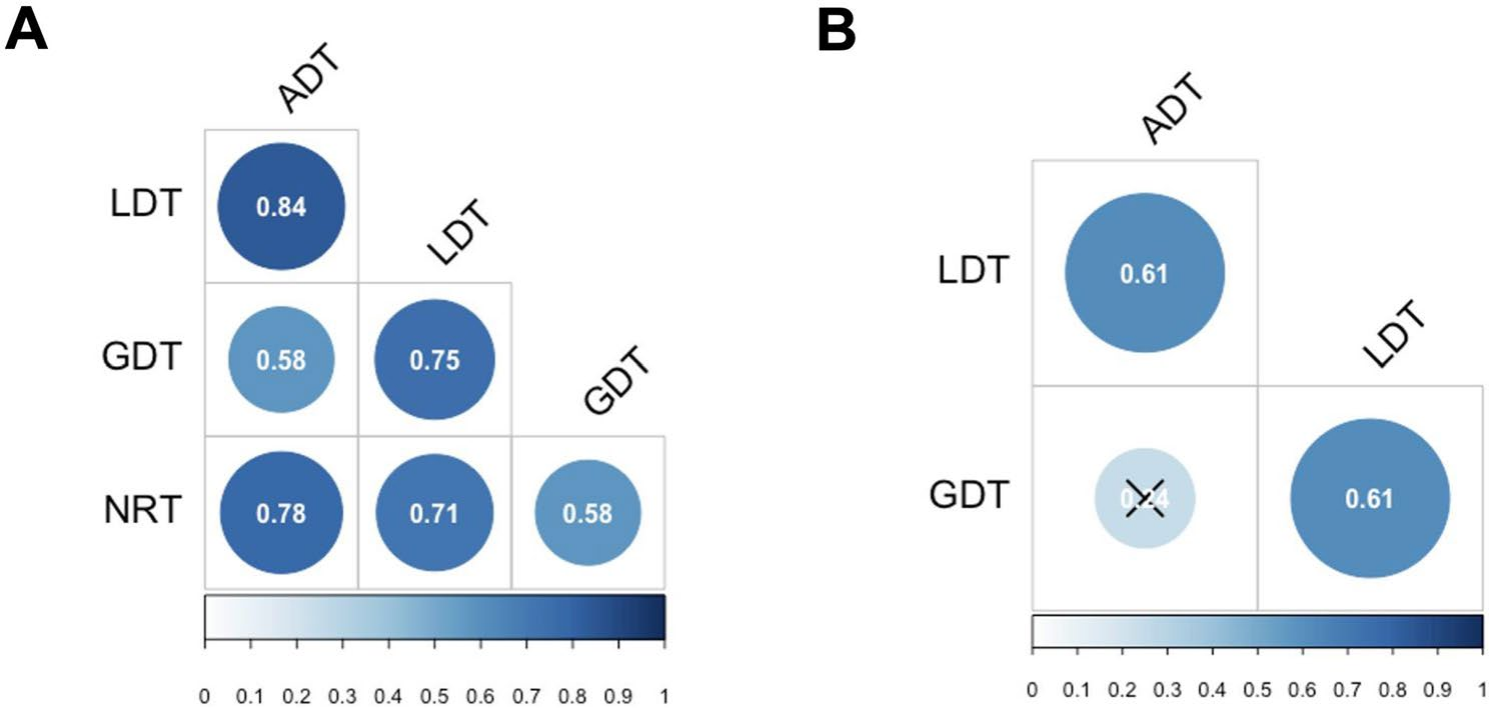
(**A**) Pairwise Pearson correlations of standardized mean RTs for correct responses by participant in each task. (**B**) Partial Pearson correlation of standardized mean RTs for correct responses by participant between ADT, LDT, and GDT while controlling for NRT (the partial correlation between GDT and ADT, marked by a cross, was not significant: p = 0.14).

Prior to analysis of RTs incorrect responses (ADT = 2.26%, LDT = 3.56%, GDT = 11.35%, and NRT = 2.03%) and correct responses with RTs less than 300 ms (ADT = 0.06%, LDT = 0.11%, GDT = 0.03%, and NRT = 0.02%) were first excluded. Then trials with outliers, defined as RTs more than 2.5 SD above or below the participant’s mean according to the type of response were excluded (ADT = 2.95%, LDT = 3.04%, GDT = 2.39%, and NRT = 3.17%). Means for correct “yes” and “no” responses per task are shown in Table 1, and the RT distributions for correct “yes” responses are shown in Fig. 2.

RTs were found to increase as the task difficulty increased and correct yes-responses were faster than correct no-responses in all tasks. Moreover, the ratio of mean RTs across tasks revealed that it takes approximately the same amount of time to produce a correct yes-response in NRT and ADT (0.93), whereas this ratio increased as the task difficulty increased (ADT vs. LDT = 1.14, LDT vs. GDT = 2.05).

### Error rates

Means of error rates for “yes” and “no” responses per task are provided in Table 1. The analysis of error rates revealed relatively few errors in NRT, ADT, and LDT (error rates below 5% for both correct yes- and no- responses), whereas larger error rates were observed in GDT (6.30% for correct yes-response, and 16.4% for correct no-response).

### Cross-task correlations on RTs

Figure 2A presents the Pearson correlations between standardized mean RTs per participant on correct yes-responses (N = 41) obtained in the different tasks computed with the *Hmisc* package in R^29^. As predicted, tasks assessing hierarchically adjacent processing showed a stronger correlation (*r*(*ADT, LDT*) = 0.83; *r*(*LDT*, *GDT*) = 0.76) than tasks assessing hierarchically distant processing (*r*(*ADT, GDT*) = 0.56). All these correlations were significant (*p* < 0.001). Moreover, NRT correlated more strongly with ADT (*r* = 0.78) and LDT (*r* = 0.72) than with GDT (*r* = 0.57). This descriptive pattern was confirmed by the analysis of the statistical difference between correlations using the *cocor* R package^30^. The difference between correlations involving adjacent levels was not statistically significant (r(ADT, LDT) = 0.83 vs. r(LDT,GDT) = 0.76, p = 0.35), whereas the differences between correlations involving non-adjacent levels were significant (r(ADT, GDT) = 0.56 vs. r(ADT, LDT) = 0.83, p < 0.001; r(ADT,GDT) = 0.56 vs. r(LDT,GDT) = 0.76, p < 0.01).

These results could be explained by the fact that the binary-decision process shared by all tasks has a higher impact on tasks where the cognitive demand is lowest as in ADT and LDT in which letter and word processing is highly automatized as compared to GDT. In order to control for the impact of this binary decision process on correlations, we computed the partial correlations between ADT, LDT, GDT while controlling for the impact of NRT. Results (see Fig. 2B) revealed that all correlations were still significant (*r*(*ADT, LDT*) = 0.61, *p* < 0.001; *r*(*LDT, GDT*) = 0.61, *p* < 0.001). However, when the binary decision component was controlled for, the correlation between non-adjacent levels of processing was no longer significant (*r*(*ADT, GDT*) = 0.24, *p* = 0.14).

### Comparison of RT distributions

**Figure 3:**
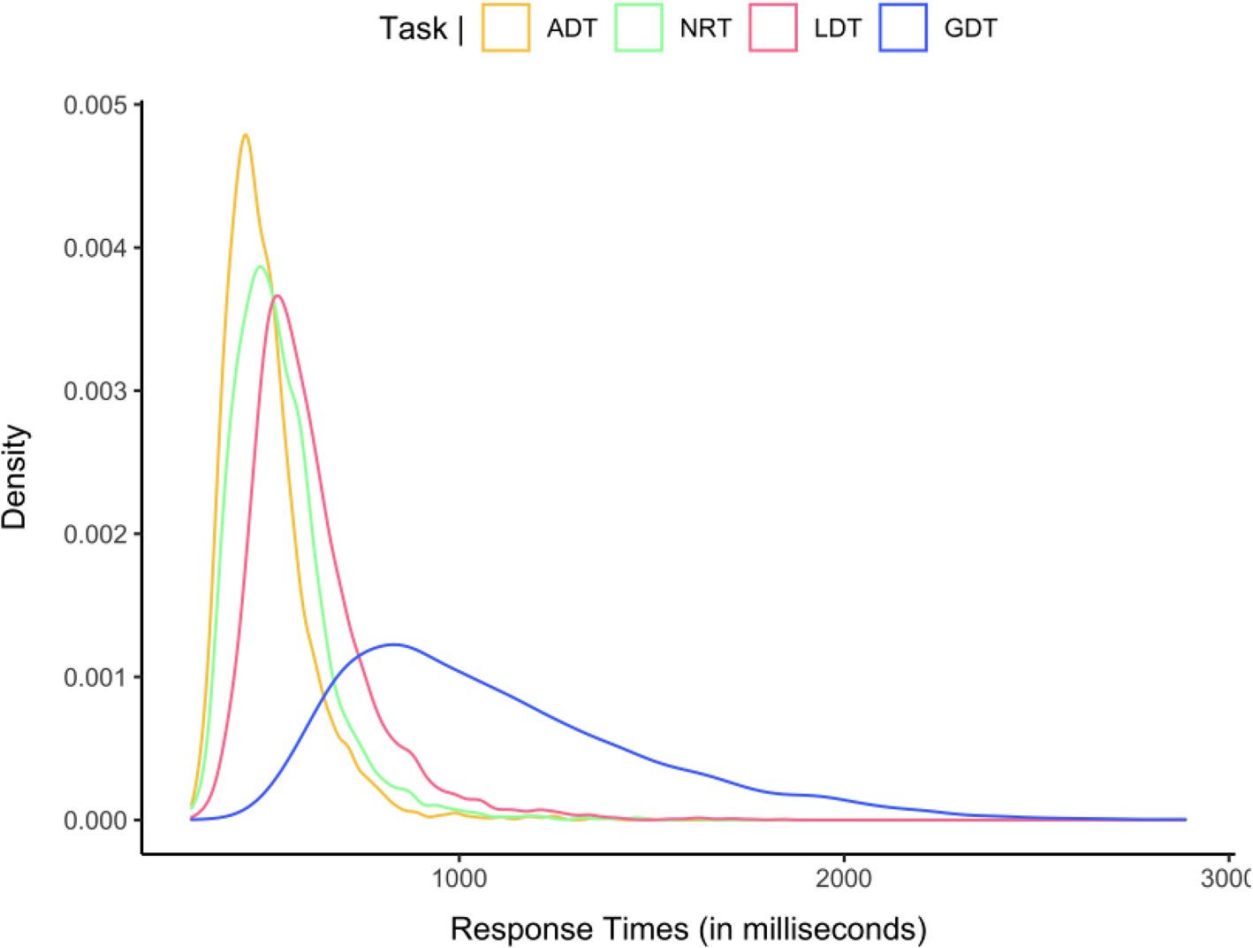
Distribution of RTs for correct “yes” responses in each task. *NRT* non-reading task, *ADT* alphabetic decision task, *LDT* lexical decision task, *GDT* grammatical decision task.

**Figure 4:**
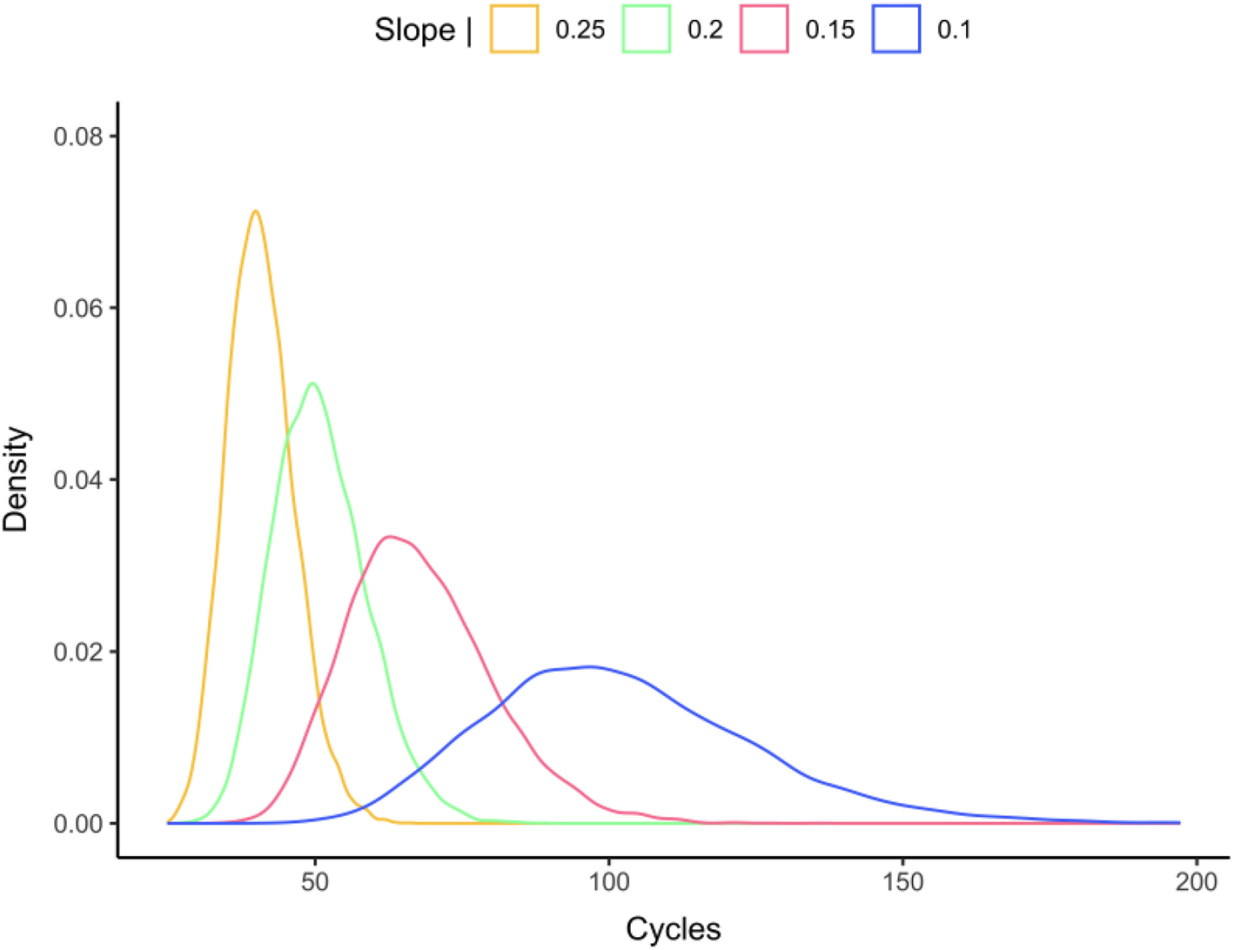
Distributions obtained with four different slopes applied to the same random walk model (starting point = 0; SD = 0.05; response criterion = 10).

In order to provide an informal demonstration that performance in the four tasks is comparable in spite of differences in average RT, in Fig. 3 we present the RT distributions for these tasks. Although the form of the distributions changed across the four tasks, we suspect that this is linked to a change in task difficulty (change in overall mean RT) that then impacts on the spread of the distributions. The most straightforward explanation for this change in RT distribution is that increasing task difficulty causes a drop in the rate of information accumulation. A slower rate of information accumulation would lead to greater mean RT as well as flatter RT distributions as seen in the empirical distributions in Fig. 3. In Fig. 4 we provide an informal proof-of-concept that this might well be the case. To do so, we generated theoretical RT distributions using a simple random walk model with a fixed decision criterion, a fixed starting point, and a fixed variance for the random walk, thus only changing the slope of the random walk (i.e., the rate of information accumulation) in four simulations. The change in rate of information accumulation was hypothesized to reflect differences in the difficulty of each task, with the slope diminishing as the task becomes harder. The theoretical RT distributions shown in Fig. 4 revealed that, with all else being equal, a decrease in slope of the random walk (from 0.25 to 0.1) mimicked the pattern seen in the empirical distributions with an increase in mean RT being associated with a flatter distribution.

## Discussion

In the present study participants performed three tasks with visual stimuli that were hypothesized to primarily reflect processing at the letter, word, and sentence levels. The tasks were the alphabetic decision task (ADT), the lexical decision task (LDT), and the grammatical decision task (GDT). A fourth non-reading task (NRT), animal/non-animal classification, was included as a baseline comparison task for speeded binary decision making involving semantic processing but with non-linguistic visual stimuli (i.e., pictures of animals and inanimate objects). Prior research, summarized in the Introduction, suggested that these three reading tasks provide a good reflection of processing at the letter, word, and sentence levels, respectively. Moreover, the three tasks are highly comparable in that they all involve making a speeded binary decision that discriminates between a given target category (i.e., a “yes” response to letters, words, or sentences, depending on the task) against a background of pseudo-stimuli from the same category (pseudo-letters, pseudo-words, and ungrammatical word sequences). We therefore reasoned that comparing performance in these tasks within the same group of participants would inform about the relations between letter, word, and sentence-level processing during reading.

An initial qualitative appraisal of processing in the three reading tasks (see Table 1) revealed, unsurprisingly, an increase in task difficulty (longer RTs and more errors for both “yes” and “no” responses) as the complexity of the task increased—from ADT, to LDT, to GDT. Performance in the non-reading task (NRT) aligned more with the ADT, and the correlation analysis revealed the same pattern (see Fig. 2). This pattern points to the addition of lexical (LDT) and sentence-level (GDT) processing on top of a speeded binary decision to simple visual stimuli as required in both the ADT and NRT.

We also compared the RT distributions in the four tasks and found that as mean RT increased there was a corresponding increase in the spread of the RT distribution (Fig. 3). We then tested the hypothesis that one key underlying mechanism at play is the rate of accumulation of information in favor of a “yes” response, which would vary as a function of task difficulty, with slower accumulation rates as the task gets harder. Simulations performed on a simple random walk model provided support for this interpretation by showing that as the slope of the random walk decreased (with all other parameters held constant) then the mean and the spread of the RT distribution increased, thus providing a qualitative match to the pattern seen in the empirical distributions (see Fig. 4).

However, the key findings of the present study concern the cross-task correlations that were found. These analyses revealed significant correlations across all tasks, albeit with weaker correlations between ADT and GDT, and between NRT and GDT. Crucially, when performance in the NRT was partialled out, the correlation between ADT and GDT was no longer significant. This absence of a correlation between non-adjacent levels of processing (i.e., letters and sentences) is clear evidence in favor of the central role for word recognition in the reading process. Moreover, without partially out performance in the NRT, statistical tests of the difference in size of correlations revealed that correlations across adjacent levels did not differ significantly, whereas the contrasts between the adjacent level correlations and the non-adjacent level correlation were significant.

The present results provide support for the simple hierarchical model of reading shown in Fig. 1, according to which word recognition plays a central role in the reading process, providing the key interface between initial letter-level processing and the final stages of sentence-level processing. Several models of text reading assign a central role to word recognition in the overall process of sentence reading (e.g., E-Z Reader^31^), and several also emphasize the critical role for orthographic processing (e.g., Glenmore^13^; OB1-reader^11^). The OB1-reader model^11^ implements the principle according to which much reading behavior can be captured by orthographic processing, implemented as the processing of letters, letter-combinations, and orthographic words (see also Grainger^16^). The present results clearly align favorably with this general approach to reading. Moreover, our results suggest that the alphabetic, lexical, and grammatical decision tasks provide a valuable window on processing at the letter, word, and sentence levels, and permit important comparisons to be made between processing (and the mechanisms driving such processing) at these different levels.

Finally, we note that one popular alternative to the hierarchical approach described in the present work, is the so-called “triangle model” of reading first proposed by Seidenberg and McClelland^32^. In this model, a non-hierarchical triangular architecture connects orthography with phonology on the one hand, and orthography with semantics on the other. One could conceivably extend this to the case of letters, words, and sentences as examined in the present work, in which case the model would predict equivalent correlations between processing times of letters and words, words and sentences, and between processing times of letters and sentences. However, it is important to stress that the key novelty introduced in the triangle model was in terms of learning mechanisms rather than mental chronometry. One could also argue that the present findings point to a common mechanism underlying processing in all three reading tasks, and one possibility here is the notion of the “quality” of representations proposed by Perfetti and Hart^33^ for lexical processing. It is possible that the same notion of “quality” could apply to both letter-level and sentence-level representations. However, this “common mechanism” approach (and the same would apply to an “individual difference” explanation—i.e., better readers are better at performing all three reading tasks) would have to explain the differences in correlations we observed between adjacent and non-adjacent levels of processing.

## Conclusions

We investigated the hierarchical nature of processing across three levels (letter, word, sentence) thought to form the backbone of reading in an alphabetic script. Participants performed three tasks, each of which was hypothesized to reflect processing at one of the three levels. When partialling out performance in a non-reading speeded binary decision task (animal vs. non-animal classification of pictures) we found significant correlations in performance in adjacent levels of processing (letter-word; word-sentence) but not between non-adjacent levels (letter-sentence). Moreover, the size of the correlations differed significantly when comparing the correlations between adjacent and non-adjacent levels, but not when comparing adjacent levels. Overall, our results point to the central role of word identification processes in mediating between lower-level sublexical processing and higher-level sentence-level processing during reading comprehension.

## Methods

### Participants

An online study consisting of three reading tasks (Alphabetical Decision, Lexical Decision, Grammatical Decision) and one non-reading task was programmed and hosted on a Labvanced server^34^.

Forty-eight participants (28 female, 20 male) were recruited via Prolific, an online platform dedicated to the recruitment of participants. Prior to the beginning of the experiment, participants were informed that data would be collected anonymously, and they provided informed consent before the experiment was initiated. The study was approved by the ethics committee of Comité de Protection des Personnes SUD-EST IV (No. 17/051). The experiment was performed in accordance with relevant guidelines and regulations and in accordance with the Declaration of Helsinki.

The order of tasks was counterbalanced across participants. Seven participants were excluded because they failed to perform at a minimum of 75% correct responses in all tasks. In addition, participants completed a questionnaire at the beginning of the study asking for age, gender, mother tongue, and handedness. Self-report for age gave a median value of 25 years (range [18; 31]). All participants reported to be native speakers of English and right-handed. The participant’s English proficiency was assessed with a computerized version of the Lextale vocabulary test^35^ delivered before the four experimental tasks (minimum of Correct Response (CR): 62%, maximum of CR: 100%, mean of CR: 88%, standard error: 9%).

### General procedure

We applied the same general procedure for all four tasks. Stimuli were displayed in black on a gray background at the center of the screen. Each trial began with a 500 ms fixation cross followed by the stimulus, which remained visible for 3000 ms or until the participant responded. Participants were asked to press the “L” key for a “yes” response and the “S” key otherwise. Then, the screen remained blank for 800 ms before the next trial. All trials were presented in a randomized order. A short practice session was proposed to the participant before the beginning of each task. The duration of each task was about 6 min for ADT, 12 min for LDT, 12 min for GDT, and 6 min for NRT, which amounted to a total of about 45 min for the entire experiment, including short breaks between each task.

### Design and stimuli

#### Alphabetic decision task (ADT)

Twenty consonant letters and 20 pseudo-letters of the Brussels Artificial Characters Sets^26^ were selected. We used the pseudo-letters from the second set (BACS-2) in which each pseudo-letter was paired with a corresponding letter according to size, number of strokes, presence/absence of symmetry, number of junctions and number of terminations. Letters were presented in Lucida Sans Unicode font and pseudo-letters in BACS-2 sans serif font. Each letter and pseudo-letter was presented three times for each of the three different sizes (size 1: 100 × 100 px, size 2: 120 × 120 px, size 3: 140 × 140 px) giving a total of 120 trials.

#### Lexical decision task (LDT)

One hundred and twenty English words were selected among those used in the grammatically correct sequences of the grammatical decision task (see next experiment section). These words were tagged as adjectives, nouns, or verbs. Due to these selection criteria, some high-frequency words used in the GDT (such as determiners, articles, prepositions) were not used in the LDT. According to the Subtlex-UK database^36^, words had a mean log-frequency of 2.17 (SD = 0.14) and a mean length of 6.05 letters (SD = 1.26 letters). Pseudowords were selected among those used in the English Lexicon Project^37^ and were matched with words on the number of letters. Stimuli were presented in 14pt Lucida Sans Unicode font.

#### Grammatical decision task (GDT)

Stimuli consisted of 240 English 4-word sequences, forming a grammatically correct structure such as “alcohol is a toxin.” The sequences were taken from the Google 4-gram English database^38^, where the term “gram” refers to a word.

Stimulus selection was operated as follows. First, we chose the 4-gram for which all words figured in the Subtlex-UK database^39^. Then, we excluded the 4-gram which contained adjectives, nouns, or verbs with less than 3 letters or more than 8 letters and whose frequency lay ± 1.75 SD beyond the average word frequency. Moreover, we ensured that the mean word lemma log10 frequency by 4-gram fell within the [1; 1] standard interval. Finally, we kept the 4-gram with Standard Frequency Index (SFI)^40^ values ranging between 1.83 and 3.83, and we removed 4-gram ending with determiners, articles, prepositions, postpositions, and particles. Two hundred and forty 4-word sequences were retained for the study. One hundred and twenty 4-gram were used as a correct grammatical sequence. The 120 remaining 4-gram were used to form ungrammatical sequences by substituting one or several words with another valid English word of the same length. This led to 120 grammatical and 120 ungrammatical sequences which were randomly presented to participants. Stimuli were presented in 14 pt Lucida Sans Unicode font.

#### Non-reading task (NRT)

Forty black and white drawings were selected from the MultiPic database^41^. MultiPic is a normative database of 750 pictures of concrete concepts dedicated for the investigation of language, visual, memory and/or attention processes. Among these 40 drawings, 20 represented a living thing (e.g., a penguin), and 20 represented a nonliving thing (e.g., an umbrella). Living and nonliving drawings were matched on all variables available in MultiPic: measures of name agreement, the percentage of valid responses, the number of different responses, the percentage of unknown responses, the percentage of idiosyncratic responses, and visual complexity. Drawings were presented three times within three different sizes (sizes were matched on the size of letter and pseudo-letters used in the alphabetic decision task), giving 120 trials.

## Data Availability

All data, materials, and code are available at the Open Science Framework (https://osf.io/a9k3g).
